# Niche dissociated assembly drives insular lizard community organization

**DOI:** 10.1038/s41598-018-30427-4

**Published:** 2018-08-10

**Authors:** Surendran Harikrishnan, Karthikeyan Vasudevan

**Affiliations:** 1 Wildlife Institute of India, Dehra Dun, Uttarakhand 248001 India; 2 Laboratory for Conservation of Endangered Species, CSIR-Centre for Cellular and Molecular Biology, Hyderabad, Telangana 500048 India

## Abstract

Interspecific competition for resources leading to niche partitioning is considered as one of the major drivers of community assembly. Competitive niche partitioning is diagnosed from species co-occurrence, species abundance distributions (SADs), and body size distributions of species. For several decades, studies have explored these patterns for the relative significance of interspecific competition in shaping communities. We explored these patterns in a finite assemblage of insectivorous lizards in the Andaman & Nicobar Islands, both at the level of archipelago and individual islands. Negative geographic co-occurrences occurred only between species pairs in islands separated by deep ocean channels. Ecologically similar species did not show positive co-occurrence in guild co-occurrence analyses, indicating that the negative geographical co-occurrences between species in islands were due to historical allopatry. Species abundance distribution was best explained by a Pareto distribution in both metacommunity and local communities. There was no predictable spacing of body sizes among co-existing species in local communities. The empirical data on insular lizard community on species co-occurrence, SADs, and body size ratios does not lend support to assortment of species in islands caused by niche subdivision. Such niche-dissociated assembly of species in islands might be an important factor in formation of biological communities, regardless of geographic scale.

## Introduction

A long-standing paradigm in ecology is the assembly of biological communities through niche partitioning among species^1,2^. For the last 50 years, ecologists have largely used the Hutchinsonian niche concept^1,3–5^, and to a lesser extent, models based on stochastic processes, to explain the assembly of communities^3–7^. It has become increasingly evident that the complexity observed in biological communities can be the result of multiple processes acting at various temporal and spatial scales. This led to a unified theory of biodiversity that applies across such temporal and spatial scales^3,4,8^. This theory assumed per capita equivalence of individuals in a community (i.e. assuming no competition) and demonstrated that biological communities could be assembled based on random ecological drift, births, and deaths^8–10^. Despite this, competitive niche division remains the most dominant paradigm used to explain the coexistence of species (or the lack of it) in ecological communities^11–15^.

Niche division resulting from competition among species is expected to produce several emergent patterns in communities. Prominent among these are: negative co-occurrence patterns, species-abundance distributions, and constant size ratios of co-existing species. At regional scales, competition theory predicts that species with similar ecological requirements (or niches) may exclude each other, and co-occur less than expected by chance. The empirical evidence for this well-studied pattern is inconclusive and inferences ambiguous^16–27^. At the local community scale, patterns in species abundance distributions (hereafter abbreviated as SAD) inform about the nature of assembly and structure of communities^1,28–32^. Though many statistical and neutral models have attempted to explain the ‘hollow curve’ of SADs, mechanistic models based on niche partitioning have dominated the discussions^9,29,32,33^. Another expectation of interspecific competition is the partitioning of body sizes among species in a community. Strong competition among similarly sized species would cause character displacement to reduce size overlap (or reduced variability in size ratios), or hamper immigration of a species having similar body size into the community^1,34–36^. Therefore, niche partitioning should produce communities in which species are less similar to each other in body size than expected by chance.

**Figure 1:**
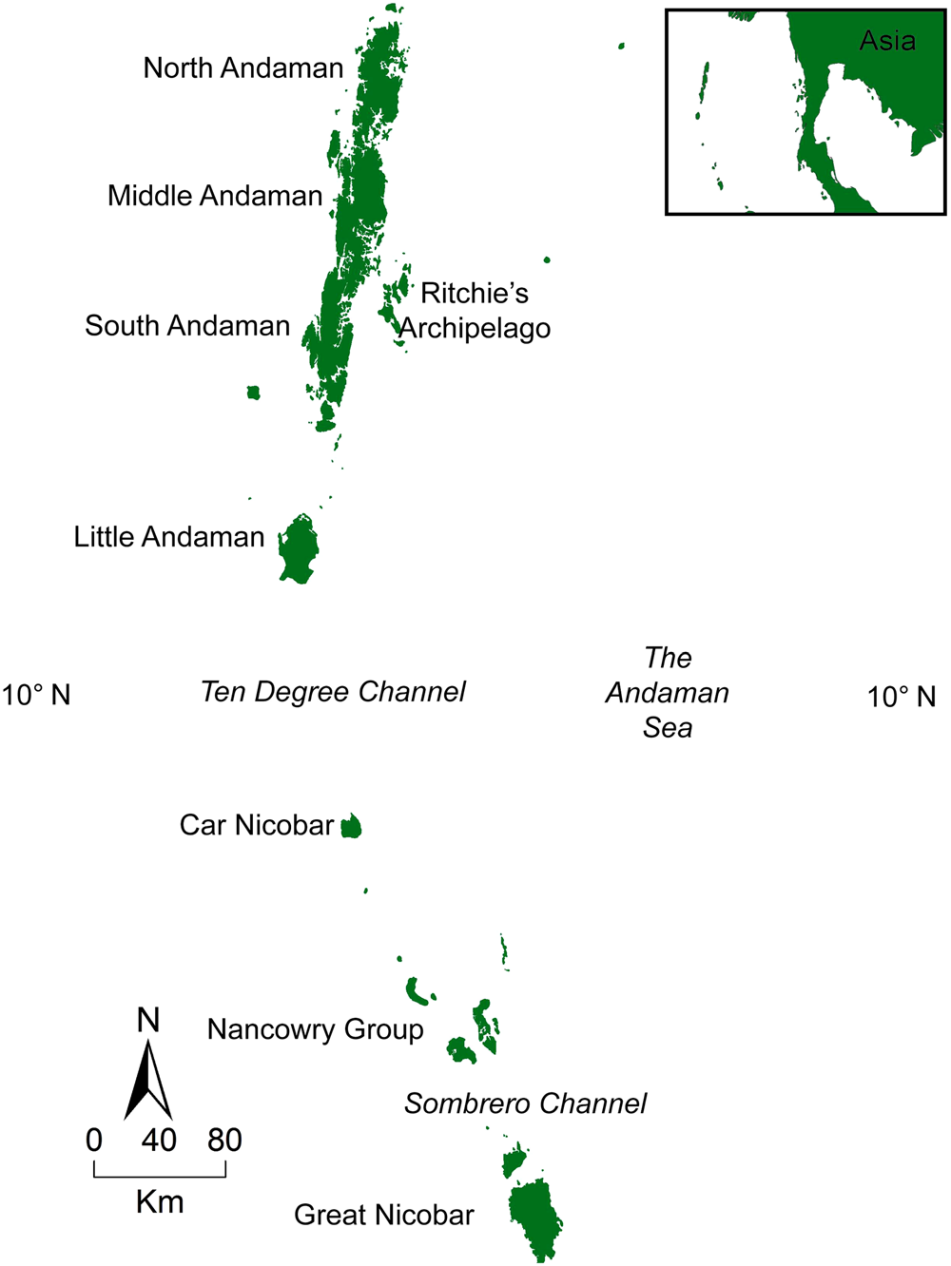
The Andaman & Nicobar Islands (ANI). The Andaman Islands (AND) are to the north of Ten Degree Channel and the Nicobar Islands (NIC) are to the south of the channel.

We tested competitive niche partitioning in a finite, indigenous, insectivorous lizard community in the Andaman & Nicobar Islands (Fig. 1), by examining patterns of species co-occurrence, SAD patterns, and body size ratios. We also examined the role of competition in structuring the lizard community at two geographic scales: (i) the archipelago or regional metacommunity and (ii) an island or local community. We used datasets on (i) species occurrences at the archipelago scale, (ii) species-abundance distributions for metacommunity and local communities, and (iii) body size ratios of co-existing species in local communities, to investigate the role of competitive niche partitioning in the assembly of this community. We show that from individual islands to archipelago, stochastic and historical factors were more important in the assembly of this insular lizard community. These findings advance our understanding of assembly of island communities, by proposing niche-dissociated processes as an important mechanism. It provides impetus for evidence-based conservation planning in the islands.

## Results

We recorded 29 out of the 31 species of indigenous insectivorous lizards known from the ANI, the remaining two species are known only from museum records. Eighteen species were endemic to ANI, with eight species endemic to AND island group, and eight to NIC island group. In bounded quadrats, we recorded 850 individuals and 14 species. We collected body mass data from 875 lizards belonging to 29 species. Similar positive species-area relationships were observed in ANI (slope z = 0.19, R^2^ = 0.66, F = 51.74, df = 27), AND (slope z = 0.20, R^2^ = 0.74, F = 33.33, df = 12), and NIC (slope z = 0.18, R^2^ = 0.51, F = 13.75, df = 13).

**Figure 2:**
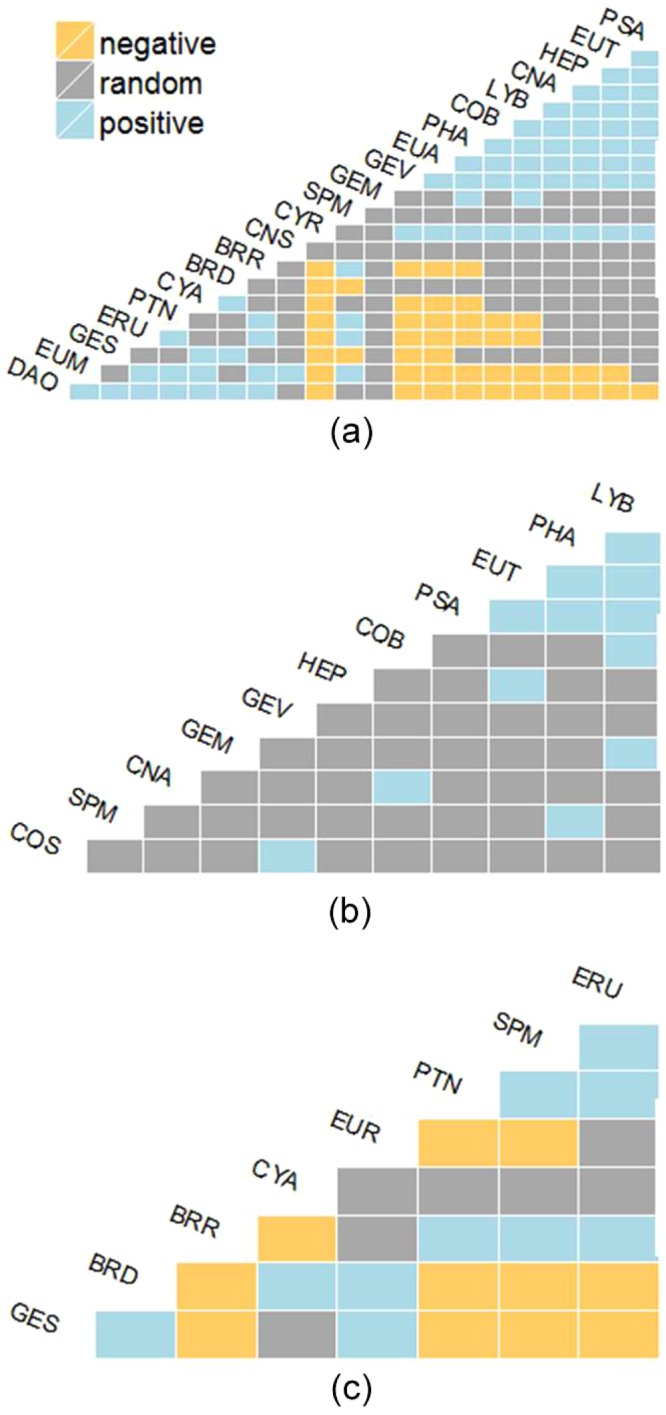
Species co-occurrence matrix for lizards in: (**a**) Andaman & Nicobar Islands (ANI) (**b**) Andaman Islands (AND) (**c**) Nicobar Islands (NIC). Species that showed only random or unclassifiable interactions are excluded from this matrix.

In ANI, nearly half of all species pairs showed non-random (either positive or negative) species co-occurrence (Fig. 2a) (Table 1). However, more species pairs showed random co-occurrence than either positive or negative interactions (Fig. 2a) (Table 1). Either the Ten Degree Channel or the smaller channels that separated the island groups in the Nicobar Islands delineated all species pairs showing negative species co-occurrence. Both AND and NIC communities had low proportions of species pairs showing non-random co-occurrence (Fig. 2b,c) (Table 1). There were no species pairs in AND that showed negative species co-occurrence (Fig. 2b) (Table 1). Only 12 pairwise interactions in AND were non-random, all of which were positive species co-occurrences (Table 1). In NIC, species pairs showed both positive and negative species co-occurrence (Fig. 2c) (Table 1). All significant negative co-occurrence occurred between pairs of species that inhabited distinct island groups within NIC (Fig. 2c). In addition, all positive co-occurrences were between species that inhabited the same island group (Fig. 2c). In both AND and NIC, the majority of interactions between species pairs were random or unclassifiable (Fig. 2b,c) (Table 1). The negative co-occurrences in ANI was due to turnover of species between AND and NIC lizard communities across the Ten Degree Channel. The probabilities of co-occurrence of individual species pairs and the standardized effect sizes in ANI, AND, and NIC are summarized in Supplementary File 5(a,b and c). *Sphenomorphus maculatus* (SPM) was the only species that occurred in both AND and NIC and showed significant co-occurrence with six other species in ANI (Fig. 2a) (Supplementary File 5a). Among these six significant co-occurrences, only two were negative – with *Bronchocela danieli* (BRD) & *Gekko smithii* (GES). Both these species occur in the southern group of Nicobar Islands, which are separated from the rest of the Nicobar Islands by the Sombrero Channel (depth > 150 m). All other species pairs that showed negative co-occurrence (shaded grey in Supplementary File 5) occurred on island groups separated by deep ocean channels, i.e., Ten Degree Channel (depth > 1000 m) and Sombrero Channel (depth > 150 m). In addition, all positive co-occurrences were between species that occurred within groups of islands separated by shallow water, such as the islands within AND separated by sea less than 50 m deep and the islands in the central group in NIC (Fig. 2). In within guild pairwise interactions, no significant pairwise interactions among species in ANI, AND, and NIC communities were observed (Table 1). Since all classified interactions in the guild co-occurrence analysis were random (Table 1), comparing the two co-occurrence analyses using a contingency table approach revealed that significant negative co-occurrences in the islands are the result of historical allopatry^37^.

**Table 1:** Summary of species co-occurrence patterns of indigenous insectivorous lizards in the Andaman and Nicobar Archipelago (ANI), the Andaman Islands (AND), and the Nicobar Islands (NIC).

Island group	Species	Sites	Positive	Negative	Random	Unclassifiable
Geographical species co-occurrence						
ANI	31	29	70	45	123	0
AND	16	14	12	0	0	16
NIC	20	15	10	11	55	5
Guild species co-occurrence						
ANI	31	4	0	0	138	327
AND	16	4	0	0	30	90
NIC	20	4	0	0	68	122

**Figure 3:**
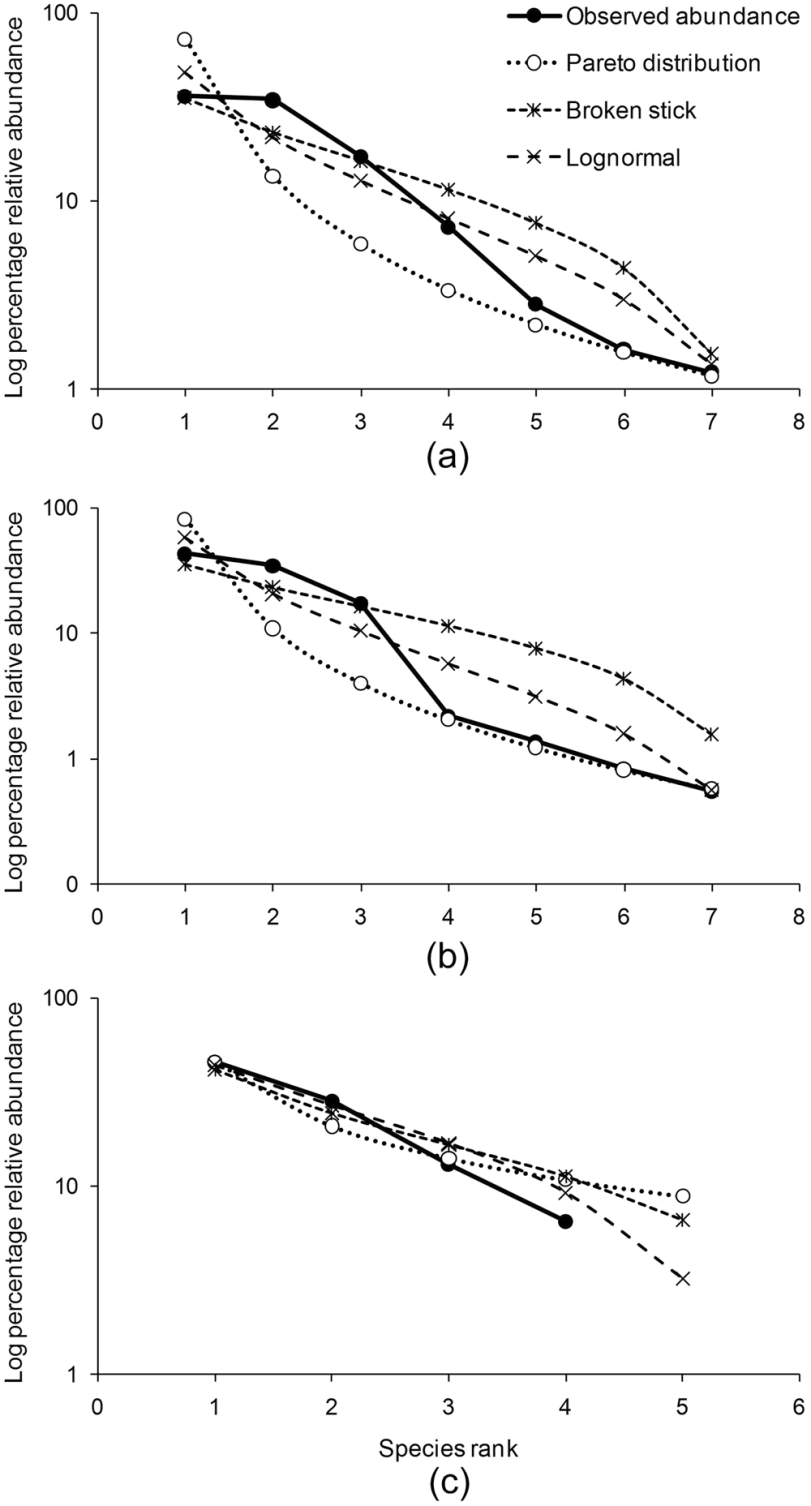
Species abundance distributions in: (**a**) Andaman Islands (AND) (**b**) Little Andaman Island (LAND) (**c**) Great Nicobar Island (GNI). Only the top three models from a ranking of models based on AIC values are presented here.

One species occurring at very high abundances and absence of rare species was characteristic of all observed SADs (Fig. 3). We examined the fit of fourteen species-abundance and relative abundance models to one metacommunity (AND) and seven local communities. Table 2 shows the top three models for AND, and two local communities (LAND and GNI) (Details of all models and communities are in Supplementary File 6). Pareto distribution ($f(x)=\frac{b{a}^{b}}{{x}^{(b+1)}}$ for all x >= scale, and f(x) = 0 otherwise, where a = scale and b = shape) fit all the island communities well, though broken-stick model (p(x) = $\frac{S-1}{N}{(1-\frac{x}{N})}^{S-2}$, where p(x) = probability density for a given abundance ‘x’, S = species richness, and N = number of individuals) also fit observed SADs for ANI, SA, RUT, NEIL, and CAM (Table 2, Supplementary File 6). Both the metacommunity and local communities exhibited similar SAD patterns (Table 2, Supplementary File 6). Examination of predicted abundance against observed values showed that pareto model best fit the observed abundance distributions. Broken-stick model fit mid-portions of the distributions (Fig. 3). Therefore, both the statistical pareto distribution and broken-stick model explained SAD patterns at different ecological scales in lizards of the Andaman & Nicobar Islands.

**Table 2:** Top three models of species abundance distribution fitted to three communities in the Andaman and Nicobar Archipelago.

SAD model	Fixed parameters	Coefficients	Estimate (±SE)	z	P	Log likelihood	df	AIC	ΔAIC
Andaman Islands (AND)									
Pareto distribution	Scale = 22	Shape	0.57 ± 0.22	2.65	8.15 × 10^−3**^	−44.72	1	91.40	0.00
Broken stick	N = 1788 S = 7	na	na	na	na	−45.79	0	91.60	0.10
Lognormal distribution	None	meanlog	4.83 ± 0.49	9.77	<2.2 × 10^−16***^	−45.65	2	95.30	3.90
		sdlog	1.31 ± 0.35	3.74	1.83 × 10^−4***^				
Little Andaman Island (LAND)									
Pareto distribution	Scale = 20	Shape	0.48 ± 0.18	2.65	8.15 × 10^−3**^	−47.80	1	97.60	0.00
Broken stick	N = 3630 S = 7	None	na	na	na	−50.98	0	102.00	4.40
Lognormal distribution	None	meanlog	5.09 ± 0.64	7.91	2.55 × 10^−15***^	−49.28	2	102.60	5.00
		sdlog	1.70 ± 0.45	3.74	1.83 × 10^−4***^				
Great Nicobar Island (GNI)									
Pareto distribution	Scale = 30	Shape	1.22 ± 0.54	2.24	0.03^*^	−25.13	1	52.30	0.00
Broken stick	N = 460 S = 5	None	na	na	na	−27.37	0	54.70	2.50
Lognormal distribution	None	meanlog	4.22 ± 0.35	12.10	<2.20 × 10^−16***^	−26.97	2	57.90	5.70
		sdlog	0.78 ± 0.25	3.16	1.57 × 10^−3**^				

The Andaman Islands (AND) lizard community is a metacommunity consisting of multiple islands in close proximity, having a common species pool and a nested structure. The Little Andaman Island (LAND) community is a local community with species belonging to the same pool as the Andaman Islands community. The Great Nicobar Island (GNI) community is a local and distinct lizard community having no shared species with the former two communities. SAD models are arranged based on descending AIC values. Details of all models in all communities are in SI 6.

Insectivorous lizards ranged in body mass from 0.5 g (*Cnemaspis* sp.) to 84 g (*Eutropis rudis*). Species richness in individual islands ranged from four to 14 species (Table 3). Observed V-ratios (variance in size ratios of co-existing lizards) varied in the 23 islands sampled from 0.15 to 13.23. There was a significant negative correlation between species richness in a community and observed V-ratios (Pearson’s product-moment correlation, R = −0.43, t = −2.17, df = 21, P = 0.04). When Chester Island, which had a large observed V-ratio (13.23) was removed from the analysis, the correlation became insignificant (R = −0.28, t = −1.33, df = 20, P = 0.20). Most islands did not show significant difference between observed and simulated V-ratios (Table 3, Supplementary File 7). In several islands, (e.g., South Andaman Island, Great Nicobar Island, Tarmugli Island, & Car Nicobar Island, see Supplementary File 7) the V-ratios expected under the null model of community assembly were similar to observed V-ratios. Since body sizes of co-existing lizards did not show constant ratios, it suggests a random assortment of species based on body sizes in the Islands.

**Table 3:** Variance ratios (V-ratio) of body sizes in lizard communities.

Islands	Species richness	Observed index	Simulated index	Variance of simulated index	Lower tail P	Upper tail P	SES
South Andaman	14	0.20	0.51	0.26	0.18	0.82	−0.61
North Andaman	12	0.34	0.81	1.68	0.28	0.72	−0.37
Long Island	11	0.24	1.04	2.02	0.07	0.93	−0.56
Neil	11	0.53	1.01	2.23	0.45	0.55	−0.32
Rutland	11	0.27	1.06	2.55	0.10	0.90	−0.50
Camorta	10	2.25	1.49	4.45	0.81	0.19	0.36
Great Nicobar	10	0.35	1.45	4.75	0.16	0.84	−0.51
Havelock	10	0.60	1.48	7.57	0.44	0.56	−0.32
Katchal	9	4.18	2.10	20.27	0.91	0.09	0.46
Car Nicobar	7	3.37	5.30	218.31	0.62	0.38	−0.13
Little Nicobar	7	1.97	5.71	584.77	0.47	0.53	−0.15
Menchal	7	0.25	5.28	169.91	0.03	0.97	−0.39
Nancowry	7	3.02	5.40	243.16	0.60	0.40	−0.15
Tarmugli	7	2.13	5.44	206.44	0.48	0.52	−0.23
Tillanchong	7	3.02	5.80	326.13	0.59	0.41	−0.15
Trinkat	7	3.02	5.63	220.58	0.60	0.40	−0.18
Teressa	7	3.02	5.03	152.10	0.60	0.40	−0.16
Bompoka	7	3.02	5.02	175.84	0.60	0.40	−0.15
Chowra	6	0.17	10.75	1448.80	0.02	0.98	−0.28
Pilo Milo	6	0.33	10.64	1398.70	0.06	0.94	−0.28
Kondul	5	0.15	20.80	9789.50	0.02	0.98	−0.21
Alexandria	4	0.88	61.50	68522.00	0.19	0.81	−0.23
Chester	4	13.23	61.72	73813.00	0.64	0.36	−0.18

We used standardized Effect Size (SES) to evaluate the significance of difference between observed and simulated V-ratios.

## Discussion

If interspecific competition influenced the probability of a species being present or absent in an island, then species pairs that showed negative geographical co-occurrence should show significant positive guild co-occurrence. We observed the highest number of pairwise negative species co-occurrences in ANI. None of these species pairs showed a significant positive guild co-occurrence. In NIC, species pairs that showed negative geographic co-occurrence did now show any positive guild co-occurrence. Therefore, we infer that negative species co-occurrences detected in ANI and NIC are the result of ‘historical allopatry’^37^. These species or their progenitors independently invaded these island groups and never came in to contact with each other. Two factors that contribute to allopatry in this case could be limited dispersal abilities of species and geographic barriers. In the case of ANI, 95% of negative co-occurrences were between species that occur on either side of the Ten Degree Channel. It is more than 1000 m deep and approximately 140 km wide and serves as a barrier maintaining historical allopatry. In AND, where almost all islands are separated by shallow sea, no significant negative co-occurrence was shown by any species pair (Figs 1 and 2b)^38–40^. Consequently, lowering of sea levels would have in the past created land connections between these islands. A few species pairs showed negative co-occurrence in NIC, where groups of islands are separated by channels shallower and narrower than the Ten Degree Channel, yet deep enough for them to be separated even during the lowest Pleistocene sea levels^38,41^. In NIC, negative co-occurrences were between species pairs occurring in islands separated by the Sombrero Channel that is more than 200 m deep (Nancowry group and the southern group of islands)^38,41^. This would have kept these island groups separated from each other even during maximum lowering of sea levels during the Pleistocene^38,41^. Therefore, we hypothesize that distribution patterns of lizards in these islands are determined by barriers to dispersal rather than interspecific competition. The positive co-occurrences opened the question of whether these are true biological associations or a result of ‘common history’^37^. On a regional scale, trait overdispersion and habitat filtering should cause species with differing ecologies to show more positive co-occurrence^42,43^. In this case, species pairs that showed positive co-occurrence in geographical matrices should have shown corresponding negative co-occurrence in the guild matrices. However, guild based co-occurrence analysis showed no significant negative interactions regardless of the geographic scale (ANI, AND, or NIC). This suggests that ecological interactions between species did not cause species to co-occur in islands. Since all the classified ecological interactions turned out to be random, the positive co-occurrences in geographical matrices can only be the result of common history^37^. In AND where most of the positive co-occurrences occurred, current species compositions in individual islands are the result of fragmentation of a larger land mass (which seemingly obtained its fauna from Southeast Asia) at the end of Pleistocene^39,40,44^.

Many recent studies have failed to detect any evidence of interspecific competition determining species co-occurrence^22,37,45–47^. A meta-analysis of several presence-absence matrices showed that in general, birds, mammals, ants, and plants showed non-random species co-occurrence while fishes, herpetofauna, and several invertebrates did not^48^. Even congeneric species – which are often thought to have similar ecological requirements and likely to exhibit negative species co-occurrence – did not exhibit non-random patterns of species co-occurrence^37,49^. Interspecific competition in insular herpetofaunal communities might not be strong enough to competitively exclude species from islands creating negative co-occurrence patterns^22^. With several macroecological datasets, including ours, now available for drawing broad generalizations, negative co-occurrence resulting in competitive niche division between closely related species is probably an exception, rather than a rule^22^.

Species abundance distributions from continents often have long negatively skewed tails composed of rare species^9^. This is missing from the Andaman & Nicobar lizard communities. In continental communities, many species are considered rare primarily due to their low detection probabilities^50^. The idea of ‘veil of rarity’ was based on the observation that several species in communities are rare and not sampled easily^51–53^. However, island communities typically have low number of species, and it is possible to sample the entire community. For terrestrial herpetofauna, bounded quadrats used for sampling reptiles provided counts of all individuals in the sampled area, thus eliminating the possibility of major differences in detection probabilities of species, which may distort patterns in relative species abundance^54–57^. Therefore, the absence of a long tail of rare species in the relative abundance distribution in this case, is not a result of insufficient sampling or imperfect detection probabilities of species in the islands. Rather, it is due to enhanced threshold probability extinction of rare species in the islands.

Explaining patterns in the abundance of species gained momentum with mechanistic models that accounted for resource partitioning by species. In this study, pareto distribution was the best SAD model consistently across all communities, though the broken-stick model also fit the metacommunity (ANI) and some of the local communities. The consistency of the fit of pareto distribution to both local communities and metacommunity showed that SAD did not change from local communities to metacommunity, exhibiting a fractal nature of islands in this archipelago. The pareto distribution is a continuous power-law density distribution that was originally used in modelling distribution of personal incomes in countries but was introduced in community ecology in an early attempt to describe SAD^58^. The similarity between distributions in non-biological systems and SAD has been pointed out earlier, but rarely been discussed in ecology^58,59^. SAD like patterns are not a unique property of ecological communities, but are common to many complex systems^60^. If ecological communities are like other non-biological complex systems, then it might be necessary to use other approaches to further explain the most commonly observed SADs^60,61^. Nekola & Brown have suggested that perhaps ecological SADs may only be explained in a post-hoc manner (e.g. examining the variation in abundances in relation to local environmental factors or other resources), and that only qualitative predictions may be possible about their nature^60^.

If body size is important in determining the niches of species, then the division of body size in communities should predict a minimum size difference between ecologically similar co-existing species^1^. Though there is not much empirical evidence for a threshold size difference^62–64^, some studies have suggested that the spacing of body sizes between co-existing species is non-random, as documented in desert rodents and local communities of bog ants^65,66^. The insectivorous lizards in the Andaman & Nicobar Islands varied in size greatly (0.5 g to 84 g). However, there was no predictable spacing of body sizes between co-existing lizard species. Our results also show that body size difference between pairs of co-existing species need not be constant either within, or between communities, of varying sizes. Stochastic species extinctions also have undoubtedly contributed to the random variation in body size differences among these lizards. These outcomes are possible when the community of lizards was assembled in the islands by passive random sampling from a metacommunity without any niche apportionment.

Ecologically similar co-existing species competing for resources and their niches differing from each other to reduce competition is a dominant paradigm in community ecology. However, we found no evidence of niche partitioning affecting the emergent properties of insular lizard communities, whether one looked at regional (species co-occurrence) or local community (SAD and size ratios) structure. Our results point at the role of historical and stochastic events in the assembly of insectivorous indigenous lizards in the Andaman & Nicobar Islands. These results, and those of several other recent studies on other taxa, downsize the role of competitive interactions among species in the assembly of communities^5,67–69^. Studies on community assembly may benefit from borrowing the methods of complexity sciences and emphasizing the recognition of potentially common underlying factors^60^.

Global lizard distribution patterns do not completely overlap with that of other vertebrates^70^. Therefore, conservation actions should take into consideration the distinctive ecological and evolutionary processes that have shaped the distribution of lizards^70^. The community of lizards that occupy the Andaman & Nicobar Islands is the outcome of processes of natural selection during their evolutionary history. Now, their persistence in these islands is fraught with human-induced habitat loss and biological invasions^71^ that will hasten their extinction. With the significant turnover of species in the archipelago (between the Andaman Islands and the Nicobar Islands; within the Nicobar Islands), any attempt to conserve species through protected areas will have to devote equal effort in all the island groups. The existing protected areas in the Andaman Islands are extensive, with over 80% of terrestrial habitats under protection. However, it is not evenly represented in all island groups. The Nancowry group, in particular, has several endemic reptiles. However, it has only one protected area in one small (~17 km^2^) island. Since geographical barriers to dispersal are the primary constraints on species distribution in the islands, conservation efforts will have to be more widespread on multiple islands and island groups.

## Methods and Materials

### Study area

The Andaman & Nicobar Islands consists of 556 islands, islets, and rocks, covering 8249 km^2^, located in the eastern part of the Bay of Bengal^44^ (Fig. 1). These islands form a continuous chain of mountains sprawling in a great arc between Cape Negrais of Myanmar and Achin Head of Sumatra, about 155 km south-east of Great Nicobar Island. It is a part of the Great Alpine-Himalayan System^41^. Paleo plate reconstructions of Southeast Asia indicate that the emergence of these islands above sea level happened only during the late Miocene (10 million years before present)^40^. While the Nicobar Islands appear to be truly oceanic in nature, surrounded by deep channels, the possibility that the Andaman Islands at their northern tip might have been connected to mainland Asia cannot be ruled out^72^. The mean annual rainfall in these islands exceeds 3000 mm, supporting a predominantly tropical evergreen vegetation^73^. The Andaman Islands are a part of the Indo-Burma biodiversity hotspot and the Nicobar Islands are part of the Sundaland biodiversity hotspot^74^. Barren and Narcondam islands are volcanic, outlying islands towards the east of the main island archipelago, and not included in this study. Many previous authors have described these islands and their fauna in detail^38,41,44,72^.

The lizard fauna of Andaman & Nicobar Islands as currently known, consists of 31 species of indigenous insectivorous lizards, one species of insectivorous/carnivorous lizard (*Varanus salvator*), and three introduced species (*Hemidactylus frenatus*, *Hemidactylus* cf. *brookii*, and *Calotes versicolor*). Only the former 31 species are considered here for further analysis as *V. salvator* occupies a different trophic level and the latter three introduced species are found only in association with human habitation. Biogeographically, the reptile fauna of the Andaman Islands has Indochinese affinities, while that of the Nicobar Islands has Malayan-Sundaland affinities^44,75^.

### Sampling lizards

We carried out intensive surveys in the Andaman & Nicobar Islands from March 2010 to January 2014, avoiding the heavy monsoon during the months of June-September. Species presence-absence was determined in 29 islands using visual encounter surveys, opportunistic records, museum records, and past publications records^44,54,76–81^. For estimating abundance, we sampled bounded quadrats of dimensions 10 m × 10 m, obtaining total counts of all individuals in the sampled quadrats^54,55,82^. We sampled 49 bounded quadrats in 14 islands the Andaman Islands (AND), with 10 of these in Little Andaman Island (LAND)^54^. In the Nicobar Islands (NIC), we sampled ten bounded quadrats in Great Nicobar Island (GNI) and four in Camorta Island (CAM). Since quadrats did not sample canopy-living species efficiently, we removed occasional records of such species from the data prior to analysis (five records out of 855 observations of lizards).

### Datasets

We recorded 29 of the 31 indigenous species of lizards occurring in the Andaman & Nicobar Islands (ANI). The remaining two species (*Scincella macrotis* and *Lipinia macrotympanum*) are known only from historical records, but with accurate locality records^78,83^. Using these 31 species, we created three ‘geographic co-occurrence’ matrices: all islands sampled in the Andaman and Nicobar Archipelago (ANI, 29 islands), the Andaman Islands (AND, 14 islands), and the Nicobar Islands (NIC, 15 islands), with species in rows and islands in columns (Supplementary File 1). AND & NIC are nested within the larger ANI community. Abbreviations for species and island names are given in Supplementary File 1. Using natural history observations and literature, we classified the lizards into four guilds based on habitat preference (Arboreal or Terrestrial) and diel activity pattern (Diurnal or Nocturnal), creating a second ‘guild co-occurrence’ matrix with species in rows and guild names in columns (Supplementary File 2).

Quadrats sampled 14 species of forest floor and understorey species from the ANI (850 individuals). Since all islands sampled in the Andaman Islands had similar lizard communities with a high degree of nestedness in species composition, we pooled this data as Andaman Islands community (AND) to estimate average abundance of a species (number of individuals/100 m^2^). For the seven individual island communities analysed – Little Andaman Island (LAND), South Andaman Island (SAND), Rutland Island (RUT), North Andaman Island (NAND), Neil Island (NEIL), Camorta Island (CAM), and Great Nicobar Island (GNI) – we estimated local abundance of lizards using only samples from these islands. Thus, we created eight data sets with species ranked in descending order of abundance (Supplementary File 3).

We collected body mass data for 29 species from 875 adult individuals (Supplementary File 4). *Scincella macrotis*, (known from specimens collected in the 19th century from a single location in Great Nicobar Island) and *Hemidactylus garnotii* were not used in analysis of body size, as we could not collect reliable size and mass information on these species. For every species, we recorded body weight in grams (W) for multiple individuals (875 individuals) using a Pesola™ Spring Balance with 0.1 g accuracy. We confirm that all methods were carried out in accordance with relevant guidelines and regulations of the Department of Environment & Forests, Andaman & Nicobar Islands Wildlife. The study was approved by the Science and Engineering Research Board, Department of Science and Technology, India. All methods were approved by the Training Research Advisory Committee of the Wildlife Institute of India.

### Data analysis

Since the islands were of varying sizes, we needed to assess potential intrinsic differences between these islands in their habitat suitability, ease of colonization, historical factors etc^84^. For this, we compared the slopes (z) of the well-known species-area relationships (the positive relationship between species richness and island areas) in ANI, AND, & NIC, using a linear least squares regression of log island area against log species richness. Since there was a high degree of overlap between communities of different islands, we assumed that any major variation in ‘z’ would be a result of intrinsic differences between islands

To explore patterns in the co-occurrence of lizards, we used a probabilistic analysis proposed by Veech^85^, by calculating the probability that two species co-occur less than or greater than the observed frequency of co-occurrence^85–87^. In a geographic co-occurrence matrix (species × islands), we counted any pair of species occurring together in the same island as a positive co-occurrence, and occurrence of only one of a pair of species in any given island as a negative co-occurrence. To examine whether dispersal limitation across biogeographical barriers would explain species co-occurrence patterns, we conducted three analyses on three presence-absence datasets: Andaman & Nicobar Islands Archipelago (ANI), the Andaman Islands (AND), and the Nicobar Islands (NIC). For ANI, we expected that the Ten Degree Channel, a deep-sea barrier between the Andaman Islands and the Nicobar Islands, would produce a significant number of negative co-occurrences purely due to limited dispersal between these groups. In AND, dispersal limitation would be a poor explanation for negative co-occurrence between species pairs as there are no major geographic barriers between the islands sampled, and interspecific interactions may be of importance^38,41^. In NIC, there are geographical barriers (channels deeper than 150 m below current sea level) between island groups (northern group, central group, and southern group)^38,41^. If negative co-occurrence patterns occur between pairs of species inhabiting different island groups in the NIC, it could be the result of dispersal limitation, while any negative co-occurrence between species inhabiting the same group could due to interspecific interactions. If competition is a causal factor, for species pairs that show significant negative geographic species co-occurrence, one would expect positive guild co-occurrence in a species × guild matrix, as competing species are expected to be ecologically similar to each other^37^.

For these analyses, we chose a minimum threshold of one for calculating expected species co-occurrence i.e., the two species may co-occur in at least one island. Species pairs that had expected co-occurrence less than one were removed from the analysis^85^. Significant positive co-occurrences are those where pairs of species co-exist in more sites than expected, while significant negative co-occurrences are those where they co-exist in fewer sites than expected (keeping α = 0.05)^85^. Random associations are those in which the observed number of co-occurrences did not deviate from their expected values by more than 0.1 times the total number of sites^85^. Standardized effect sizes were calculated as observed – expected value divided by total number of sites (range −1 to 1)^85^. We first analysed pairwise geographic species co-occurrence (species × island). To ascertain the causal factors behind the observed patterns, we conducted a second set of analyses on guild species co-occurrence for ANI, AND, & NIC (species × guild)^37^. The results of geographical and guild co-occurrence analyses were compared using an interaction matrix proposed by Sfenthourakis^37^. All analyses were conducted in programme R using the package ‘cooccur’^87,88^. Of the 465 species pair combinations in ANI, we removed 227 pairs (48.82%) from the analysis because their expected co-occurrence was <1. Similarly, we removed 27 (22.5%) out of 120 pairs in AND and 109 (57.37%) out of 190 pairs in NIC from the analysis. Thus, we analyzed 238 species pairs in ANI, 93 in AND, and81 in NIC.

We examined species abundance distributions (SAD) by fitting fourteen well-known models of species-abundance/rank-abundance to observed data using a Maximum Likelihood Estimation based fitting procedure^88,89^. The models examined here are: pareto distribution^90^, broken-stick model^30^, geometric series^91^, log-series^92^, lognormal^51^, Weibull distribution^93^, Power-discreet distribution^90^, Zipf distribution^90^, Zipf-Mandelbrot distribution^94^, zero-sum multinomial distribution^95^, poisson lognormal^96^, neutral model^97^, negative binomial^98^, and gamma distribution^99^. We used Akaike Information Criteria (AIC) for ranking the models in descending order. We examined SADs for terrestrial lizards in the Andaman Islands (AND) which is a metacommunity, and seven local communities (individual islands) – LAND, SAND, RUT, NAND, NEIL, CAM, and GNI (for abbreviations, see Annexure 1). These analyses were conducted in programme R, using the package ‘SADs’^88,89^.

Ordered, log transformed body mass data was used to examine whether lizard communities exhibited a constant size ratio. We used the variance in size ratios (V-ratio) as a metric to examine the distribution of body sizes among species in 23 local communities (individual islands)^100^. We looked for non-random size ratios in these communities by comparing them with expected V-ratios in null communities having equal species richness. To create null communities, we first defined a source pool consisting of all species of insectivorous lizards in the ANI. We added an arbitrary five hypothetical species to this source pool to ensure that the source pool community always had more species than all local communities did. We kept the maximum body size in the source community identical to the largest body size exhibited by the real community. We drew null communities from this source pool and compared the V-ratios against observed local communities. We used Standardized Effect Size (SES) to evaluate the significance of difference between observed and simulated V-ratios. These analyses were performed using the R package EcosimR^88,101^.

## Electronic supplementary material

Supplementary information
