# Supplementary material for: Niche dissociated assembly drives insular lizard community organization
Source: Sci Rep. 2018 Aug 10;8:11978. doi: 10.1038/s41598-018-30427-4 (PMC6086863; doi:10.1038/s41598-018-30427-4)

Niche dissociated assembly drives insular lizard community organization

Surendran Harikrishnan[1] & Karthikeyan Vasudevan[*2]

[1] – Wildlife Institute of India, Dehra Dun, Uttarakhand, India – 248001

[2] – Laboratory for Conservation of Endangered Species, CSIR- Centre for Cellular and Molecular Biology, Hyderabad, Telangana, India – 500048 E-mail: karthik@ccmb.res.in

Supplementary Files

Supplementary File 1. Presence-absence matrix of indigenous insectivorous lizards in the Andaman & Nicobar Archipelago, with species names in rows and island names in columns. Human introduced species are excluded from this list. The last row summarizes species richness (S) in each island. Abbreviations for islands: CAN – Car Nicobar, CHO – Chowra, BOM – Bompoka, TIL – Tillangchong, TER – Terassa, TRI – Trinkat, CAM – Camorta, NAN – Nancowry, KAT – Katchall, PIM – Pilo Milo, LIN – Little Nicobar, MEN – Menchal, PIG – Pigeon, GNI – Great Nicobar, SAND – South Andaman, NAND – North Andaman, LAND – Little Andaman, RUT – Rutland, HAV – Havelock, LON – Long Island, NEI – Neil Island, TAR – Tarmugli, ALE – Alexandra, HOB – Hobday, RED – Redskin, SNO – Snob Island, CHE – Chester Island, GRU – Grub Island.

Abbreviations for species names: BRC – *Bronchocela cristatella*, BRD – *Bronchocela danieli*, BRR – *Bronchocela rubrigularis*, CNS – *Cnemaspis* sp.2, COS – *Coryphophylax subcristatus*, CYA – *Cyrtodactylus adleri*, DAN – *Dasia nicobarensis*, DAO – *Dasia olivacea*, DIN – *Dibamus nicobaricum*, GES – *Gekko smithii*, HEG – *Hemidactylus garnotii*, HET – *Hemiphyllodactylus typus*, LIM – *Lipinia macrotympanum*, EUM – *Eutropis multifasciata*, EUR – *Eutropis rudis*, ERU – *Eutropis rugifera*, PTN – *Ptychozoon nicobarensis*, SCM – *Scincella macrotis*, SPM – *Sphenomorphus maculatus*, PSA – *Pseudocalotes andamanensis*, CNA – *Cnemaspis* sp.1, COB – *Coryphophylax brevicaudus*, CYR – *Cyrtodactylus rubidus*, GEM – *Gehyra mutilata*, GEV – *Gekko verreauxi*, HEP – *Hemidactylus platyurus*, LEL – *Lepidodactylus lugubris*, LYB – *Lygosoma bowringii*, EUA – *Eutropis andamanensis*, EUT – *Eutropis tytleri*, PHA – *Phelsuma andamanense*.

	Nicobar Islands															Andaman Islands													
	CAN	CHO	BOM	TIL	TER	TRI	CAM	NAN	KAT	PIM	LIN	MEN	KON	PIG	GNI	SAND	NAND	LAND	RUT	HAV	LON	NEI	TAR	ALE	HOB	RED	SNO	CHE	GRU
**BRC**	1	0	0	0	0	0	0	0	0	0	0	0	0	0	0	0	0	0	0	0	0	0	0	0	0	0	0	0	0
**BRD**	0	0	0	0	0	0	0	0	0	1	1	1	1	0	1	0	0	0	0	0	0	0	0	0	0	0	0	0	0
**BRR**	0	1	1	1	1	1	1	1	1	0	0	0	0	0	0	0	0	0	0	0	0	0	0	0	0	0	0	0	0
**CNS**	0	0	0	0	0	0	1	0	1	0	0	0	0	0	1	0	0	0	0	0	0	0	0	0	0	0	0	0	0
**COS**	1	1	1	1	1	1	1	1	1	1	1	1	1	0	0	1	1	1	1	1	1	1	1	1	1	1	0	0	0

**CYA**	1	0	0	0	0	0	1	0	1	1	1	1	1	0	1	0	0	0	0	0	0	0	0	0	0	0	0	0	0
**DAN**	1	0	0	0	0	0	0	0	0	0	0	0	0	0	0	0	0	0	0	0	0	0	0	0	0	0	0	0	0
**DAO**	0	1	1	1	1	1	1	1	1	1	1	1	1	0	1	0	0	0	0	0	0	0	0	0	0	0	0	0	0
**DIN**	0	0	0	0	0	0	0	0	0	0	0	0	0	0	1	0	0	0	0	0	0	0	0	0	0	0	0	0	0
**GES**	0	0	0	0	0	0	0	0	0	1	1	1	1	1	1	0	0	0	0	0	0	0	0	0	0	0	0	0	0
**HEG**	0	0	0	0	0	1	0	0	0	0	0	0	0	0	0	0	0	0	0	0	0	0	0	0	0	0	0	0	0
**HET**	0	0	0	0	0	0	0	0	0	0	0	0	0	0	1	1	0	0	0	0	1	0	0	0	0	0	0	0	0
**LIM**	0	0	0	0	0	0	0	0	0	0	1	0	0	0	1	1	0	0	0	0	0	0	0	0	0	0	0	0	0
**EUM**	1	0	1	1	1	1	1	1	1	0	1	1	0	0	1	0	0	0	0	0	0	0	0	0	0	0	0	0	0
**EUR**	0	0	0	0	0	0	0	0	0	1	0	1	0	0	1	0	0	0	0	0	0	0	0	0	0	0	0	0	0
**ERU**	0	1	1	1	1	1	1	1	1	0	0	0	0	0	1	0	0	0	0	0	0	0	0	0	0	0	0	0	0
**PTN**	1	1	1	1	1	1	1	1	1	0	0	0	0	0	0	0	0	0	0	0	0	0	0	0	0	0	0	0	0
**SCM**	0	0	0	0	0	0	0	0	0	0	0	0	0	0	1	0	0	0	0	0	0	0	0	0	0	0	0	0	0
**SPM**	1	1	1	1	1	1	1	1	1	0	0	0	0	0	0	1	1	0	1	1	0	0	0	0	0	0	0	1	0
**PSA**	0	0	0	0	0	0	0	0	0	0	0	0	0	0	0	1	1	1	1	0	1	0	0	0	0	0	0	0	0
**CNA**	0	0	0	0	0	0	0	0	0	0	0	0	0	0	0	1	1	1	0	1	0	1	1	0	0	0	0	0	0
**COB**	0	0	0	0	0	0	0	0	0	0	0	0	0	0	0	1	1	1	1	1	0	1	1	0	0	0	0	0	0
**CYR**	0	0	0	0	0	0	0	0	0	0	0	0	0	0	0	1	1	1	1	1	1	1	1	1	1	1	1	1	1
**GEM**	0	0	0	0	0	0	1	0	0	0	0	0	0	0	1	1	0	0	0	1	1	1	0	0	0	0	0	0	0
**GEV**	0	0	0	0	0	0	0	0	0	0	0	0	0	0	0	1	1	1	1	1	1	1	1	1	1	1	0	0	0

**HEP**	0	0	0	0	0	0	0	0	0	0	0	0	0	0	0	1	1	0	1	0	1	1	1	0	0	0	0	0	0
**LEL**	0	0	0	0	0	0	0	0	0	0	0	0	0	0	0	1	0	1	0	0	0	0	0	0	0	0	0	0	0
**LYB**	0	0	0	0	0	0	0	0	0	0	0	0	0	0	0	1	1	1	1	1	1	1	0	0	0	0	0	0	0
**EUA**	0	0	0	0	0	0	0	0	0	0	0	0	0	0	0	1	1	1	1	1	1	1	1	1	0	1	0	1	1
**EUT**	0	0	0	0	0	0	0	0	0	0	0	0	0	0	0	1	1	1	1	0	1	1	0	0	0	0	0	0	0
**PHA**	0	0	0	0	0	0	0	0	0	0	0	0	0	0	0	1	1	1	1	1	1	1	0	0	0	0	0	1	0
S	**6**	**6**	**7**	**7**	**7**	**8**	**8**	**8**	**7**	**5**	**6**	**6**	**4**	**1**	**13**	**16**	**12**	**11**	**11**	**10**	**11**	**11**	**7**	**4**	**3**	**4**	**1**	**4**	**2**

Supplementary File 2. Ecological co-occurrence matrix for indigenous insectivorous lizards in the Andaman & Nicobar Archipelago, with species names in rows and ecological guilds in columns. Abbreviations for species names are given in Annexure 1.

	Arboreal	Terrestrial	Diurnal	Nocturnal
**10**	1	0	1	0
**BRD**	1	0	1	0
**BRR**	1	0	1	0
**CNS**	1	1	1	0
**COS**	1	1	1	0
**CYA**	1	1	0	1
**DAN**	1	0	1	0
**DAO**	1	0	1	0
**DIN**	0	1	1	0
**GES**	1	0	0	1
**HEG**	1	0	0	1
**HET**	0	1	0	1
**LIM**	0	1	1	0
**EUM**	0	1	1	0
**EUR**	0	1	1	0
**ERU**	0	1	1	0
**PTN**	1	0	0	1
**SCM**	0	1	1	0
**SPM**	0	1	1	0
**PSA**	1	0	1	0
**CNA**	1	1	1	0
**COB**	1	1	1	0
**CYR**	1	1	0	1
**GEM**	1	0	0	1
**GEV**	1	0	0	1
**HEP**	1	0	0	1
**LEL**	1	0	0	1
**LYB**	0	1	1	0
**EUA**	0	1	1	0
**EUT**	1	1	1	0
**PHA**	1	0	1	0

Supplementary File 3. Abundance of forest floor and understorey lizards recorded in bounded quadrats in the Andaman & Nicobar Islands. Abundance was estimated as number of individuals/100m^2 (±SE), but for analyses, they were converted to individuals/ha. (na – Not recorded in quadrats in that island).

Species	Andaman Islands (ANI)	South Andaman (SAND)	Little Andaman (LAND)	Rutland Island (RUT)	North Andaman (NAND)	Neil Island (NEIL)	Camorta Island (CAM)	Great Nicobar (GNI)
Coryphophylax subcristatus	6.40±0.71	8.88±1.26	12.60±1.88	8.20±2.58	3.25±0.75	4.00±1.00	5.50±1.20	na
Lygosoma bowringii	6.12±1.79	0.38±0.18	15.60±4.11	1.20±1.20	0.50±0.29	15.00±9.00	na	na
Cyrtodactylus rubidus	3.06±0.46	2.13±0.64	6.30±1.56	1.80±0.58	3.50±0.65	6.50±2.50	na	na
Coryphophylax brevicaudus	1.29±0.32	2.25±0.88	0.50±0.27	1.60±0.68	0.75±0.48	0.50±0.50	na	na
Cnemaspis sp.1	0.50±0.22	0.38±0.26	0.80±0.51	na	na	na	na	na
Eutropis andamanensis	0.29±0.08	0.13±0.13	0.30±0.15	0.40±0.40	0.75±0.48	1±0	na	na
Sphenomorphus maculatus	0.22±0.10	na	0.20±0.20	na	0.50±0.50	na	na	na
Cnemaspis sp.2	na	na	na	na	na	na	na	2.10±0.67
Eutropis rugifera	na	na	na	na	na	na	0.50±0.30	1.30±0.50
Cyrtodactylus adleri	na	na	na	na	na	na	1.25±0.50	0.60±0.31
Dibamus nicobaricus	na	na	na	na	na	na	na	0.30±0.21
Eutropis rudis	na	na	na	na	na	na	na	0.30±0.15
Eutropis multifasciata	na	na	na	na	na	na	na	na
Gehyra mutilata	na	na	na	na	na	na	0.25±0.30	na

Supplementary File 4. Average snout-vent length (SVL) and body masses (W) of indigenous insectivorous lizards in the Andaman & Nicobar Archipelago. N is the number of individuals measured. For *Lipinia macrotympana* only two museum specimens were measured.

Species	SVL ± SD (mm)	W ± SD (g)	N
Bronchocela cristatella	90.47 ± 7.16	12.55 ± 2.48	5
Bronchocela danieli	74.12 ± 7.25	6.66 ± 1.82	12
Bronchocela rubrigularis	88.88 ± 10.84	11.88 ± 3.66	5
Cnemaspis sp.1	26.71 ± 6.07	0.48 ± 0.26	6
Cnemaspis sp. 2	30.96 ± 3.21	0.62 ± 0.08	5
Coryphophylax brevicaudus	57.27 ± 6.72	4.61 ± 1.39	51
Coryphophylax subcristatus	71.38 ± 14.68	9.46 ± 5.71	382
Cyrtodactylus adleri	56.00 ± 11.50	3.61 ± 1.58	12
Cyrtodactylus rubidus	52.87 ± 14.04	3.15 ± 2.13	108
Dasia nicobarensis	88.51 ± 5.58	12.93 ± 0.78	8
Dasia olivacea	85.65 ± 2.83	13.55 ± 1.15	11
Dibamus nicobaricus	119.25 ± 25.12	1.71 ± 0.87	8
Eutropis andamanensis	98.95 ± 26.36	32.24 ± 19.82	11
Eutropis multifasciata	130.37 ± 18.53	73.93 ± 25.67	8
Eutropis rudis	137.73 ± 6.36	84.12 ± 10.99	6
Eutropis rugifera	54.95 ± 6.52	4.63 ± 1.52	9
Eutropis tytleri	151.16 ± 4.14	82.77 ± 3.03	7
Gehyra mutilata	42.16 ± 5.49	2.16 ± 0.80	9
Gekko smithii	126.47 ± 2.80	30.33 ± 2.57	3
Gekko verreauxi	145.81 ± 6.52	52.06 ± 8.78	7
Hemidactylus platyurus	48.97 ± 5.82	2.40 ± 0.63	4
Hemiphyllodactylus typus	32.65 ± 7.25	0.80 ± 0.30	6
Lepidodactylus lugubris	36.45 ± 1.80	1.35 ± 0.05	5
Lipinia macrotympana	37.30 ± 1.13	0.70	2
Lygosoma bowringii	41.48 ± 5.60	1.25 ± 0.32	152
Phelsuma andamanense	55.63 ± 3.16	4.25 ± 0.49	8
Pseudocalotes andamanensis	82.25 ± 6.08	11.85 ± 1.32	6
Ptychozoon nicobarensis	91.42 ± 6.24	11.20 ± 1.82	3
Sphenomorphus maculatus	56.56 ± 7.02	4.31 ± 1.45	8

Supplementary File 5a. Summary of significant co-occurrence pairs in the Andaman & Nicobar Archipelago (ANI). Obs.cooc – observed number of co-occurrences; Prob.cooc – probability of co-occurrence; Exp.cooc – expected number of co-occurrences; p_{lt}– probability that the two species would co-occur at a frequency less than the observed number of co-occurrence sites if the two species were distributed randomly (independently) of one another; p_{gt} – probability of co-occurrence at a frequency greater than the observed frequency; Std.eff – standardized effect size. Shaded species pairs show significant negative co-occurrences.

No. sites with Sp1	No. sites with Sp2	Obs. cooc.	Prob. cooc.	Exp. cooc.	p_{lt}	p_{gt}	Std.eff	Sp1 name	Sp2 name
5	8	5	0.048	1.4	1	0.001	0.12	BRD	CYA
5	13	5	0.077	2.2	1	0.011	0.10	BRD	DAO
5	6	5	0.036	1	1	5×10^{-5}	0.14	BRD	GES
5	14	0	0.083	2.4	0.025	1	-0.08	BRD	SPM
5	14	0	0.083	2.4	0.025	1	-0.08	BRD	CYR
8	13	8	0.124	3.6	1	3×10^{-4}	0.15	BRR	DAO
8	11	7	0.105	3	0.999	0.001	0.14	BRR	EUM
8	9	8	0.086	2.5	1	0	0.19	BRR	ERU
8	9	8	0.086	2.5	1	0	0.19	BRR	PTN
8	14	8	0.133	3.9	1	7×10^{-4}	0.14	BRR	SPM
8	14	0	0.133	3.9	0.002	1	-0.13	BRR	CYR
8	11	0	0.105	3	0.010	1	-0.10	BRR	GEV
8	12	0	0.114	3.3	0.006	1	-0.11	BRR	EUA
8	8	0	0.076	2.2	0.047	1	-0.08	BRR	PHA
3	11	3	0.039	1.1	1	0.045	0.07	CNS	EUM
8	13	7	0.124	3.6	0.999	0.007	0.12	CYA	DAO
8	6	5	0.057	1.7	0.999	0.003	0.11	CYA	GES
8	11	6	0.105	3	0.999	0.018	0.10	CYA	EUM
8	14	0	0.133	3.9	0.002	1	-0.13	CYA	CYR
8	11	0	0.105	3	0.010	1	-0.10	CYA	GEV
8	12	0	0.114	3.3	0.006	1	-0.11	CYA	EUA
8	8	0	0.076	2.2	0.047	1	-0.08	CYA	PHA
13	6	5	0.093	2.7	0.996	0.047	0.08	DAO	GES
13	11	10	0.170	4.9	1	1×10^{-4}	0.18	DAO	EUM
13	9	9	0.139	4	1	7×10^{-5}	0.17	DAO	ERU
13	9	8	0.139	4	0.999	0.002	0.14	DAO	PTN
13	5	0	0.077	2.2	0.037	1	-0.08	DAO	PSA
13	6	0	0.093	2.7	0.017	1	-0.09	DAO	CNA
13	7	0	0.108	3.1	0.007	1	-0.11	DAO	COB
13	14	0	0.216	6.3	0	1	-0.22	DAO	CYR
13	11	0	0.170	4.9	1×10^{-4}	1	-0.17	DAO	GEV
13	6	0	0.093	2.7	0.017	1	-0.09	DAO	HEP
13	7	0	0.108	3.1	0.007	1	-0.11	DAO	LYB
13	12	0	0.185	5.4	4×10^{-5}	1	-0.19	DAO	EUA

13	6	0	0.093	2.7	0.017	1	-0.09	DAO	EUT
13	8	0	0.124	3.6	0.003	1	-0.12	DAO	PHA
6	14	0	0.100	2.9	0.011	1	-0.10	GES	SPM
6	14	0	0.100	2.9	0.011	1	-0.10	GES	CYR
6	11	0	0.078	2.3	0.039	1	-0.08	GES	GEV
6	12	0	0.086	2.5	0.026	1	-0.09	GES	EUA
11	9	8	0.118	3.4	0.999	3×10^{-4}	0.16	EUM	ERU
11	9	8	0.118	3.4	0.999	3×10^{-4}	0.16	EUM	PTN
11	14	8	0.183	5.3	0.994	0.046	0.09	EUM	SPM
11	6	0	0.078	2.3	0.039	1	-0.08	EUM	CNA
11	7	0	0.092	2.7	0.02	1	-0.09	EUM	COB
11	14	0	0.183	5.3	4×10^{-5}	1	-0.18	EUM	CYR
11	11	0	0.144	4.2	0.001	1	-0.14	EUM	GEV
11	6	0	0.078	2.3	0.039	1	-0.08	EUM	HEP
11	7	0	0.092	2.7	0.020	1	-0.09	EUM	LYB
11	12	0	0.157	4.6	4×10^{-4}	1	-0.16	EUM	EUA
11	6	0	0.078	2.3	0.039	1	-0.08	EUM	EUT
11	8	0	0.105	3	0.010	1	-0.10	EUM	PHA
9	9	8	0.096	2.8	1	2×10^{-5}	0.18	ERU	PTN
9	14	8	0.150	4.3	0.999	0.005	0.13	ERU	SPM
9	7	0	0.075	2.2	0.050	1	-0.08	ERU	COB
9	14	0	0.150	4.3	5×10^{-4}	1	-0.15	ERU	CYR
9	11	0	0.118	3.4	0.005	1	-0.12	ERU	GEV
9	7	0	0.075	2.2	0.050	1	-0.08	ERU	LYB
9	12	0	0.128	3.7	0.002	1	-0.13	ERU	EUA
9	8	0	0.086	2.5	0.029	1	-0.09	ERU	PHA
9	14	9	0.150	4.3	1	2×10^{-4}	0.16	PTN	SPM
9	7	0	0.075	2.2	0.050	1	-0.08	PTN	COB
9	14	0	0.150	4.3	5×10^{-4}	1	-0.15	PTN	CYR
9	11	0	0.118	3.4	0.005	1	-0.12	PTN	GEV
9	7	0	0.075	2.2	0.050	1	-0.08	PTN	LYB
9	12	0	0.128	3.7	0.002	1	-0.13	PTN	EUA
9	8	0	0.086	2.5	0.029	1	-0.09	PTN	PHA
5	6	3	0.036	1	0.997	0.046	0.07	PSA	CNA
5	7	4	0.042	1.2	0.999	0.007	0.10	PSA	COB
5	14	5	0.083	2.4	1	0.017	0.09	PSA	CYR
5	11	5	0.065	1.9	1	0.004	0.11	PSA	GEV
5	6	4	0.036	1	0.999	0.003	0.10	PSA	HEP
5	7	5	0.042	1.2	1	1×10^{-4}	0.13	PSA	LYB
5	12	5	0.071	2.1	1	0.007	0.10	PSA	EUA
5	6	5	0.036	1	1	5×10^{-5}	0.14	PSA	EUT
5	8	5	0.048	1.4	1	0.001	0.12	PSA	PHA
6	7	6	0.050	1.4	1	1×10^{-5}	0.16	CNA	COB
6	14	6	0.100	2.9	1	0.006	0.11	CNA	CYR
6	11	6	0.078	2.3	1	0.001	0.13	CNA	GEV
6	6	4	0.043	1.2	0.999	0.008	0.10	CNA	HEP

6	7	5	0.050	1.4	0.999	0.001	0.12	CNA	LYB
6	12	6	0.086	2.5	1	0.002	0.12	CNA	EUA
6	6	4	0.043	1.2	0.999	0.008	0.10	CNA	EUT
6	8	5	0.057	1.7	0.999	0.003	0.11	CNA	PHA
7	14	7	0.117	3.4	1	0.002	0.12	COB	CYR
7	11	7	0.092	2.7	1	2×10^{-4}	0.15	COB	GEV
7	6	5	0.050	1.4	0.999	0.001	0.12	COB	HEP
7	7	6	0.058	1.7	1	1×10^{-4}	0.15	COB	LYB
7	12	7	0.100	2.9	1	0.001	0.14	COB	EUA
7	6	5	0.050	1.4	0.999	0.001	0.12	COB	EUT
7	8	6	0.067	1.9	0.999	4×10^{-4}	0.14	COB	PHA
14	11	11	0.183	5.3	1	1×10^{-5}	0.20	CYR	GEV
14	6	6	0.100	2.9	1	0.006	0.11	CYR	HEP
14	7	7	0.117	3.4	1	0.002	0.12	CYR	LYB
14	12	12	0.200	5.8	1	0	0.21	CYR	EUA
14	6	6	0.100	2.9	1	0.006	0.11	CYR	EUT
14	8	8	0.133	3.9	1	7×10^{-4}	0.14	CYR	PHA
6	7	4	0.050	1.4	0.999	0.018	0.09	GEM	LYB
6	8	4	0.057	1.7	0.998	0.034	0.08	GEM	PHA
11	6	6	0.078	2.3	1	0.001	0.13	GEV	HEP
11	7	7	0.092	2.7	1	2×10^{-4}	0.15	GEV	LYB
11	12	10	0.157	4.6	1	3×10^{-5}	0.19	GEV	EUA
11	6	6	0.078	2.3	1	0.001	0.13	GEV	EUT
11	8	7	0.105	3	0.999	0.001	0.14	GEV	PHA
6	7	5	0.050	1.4	0.999	0.001	0.12	HEP	LYB
6	12	6	0.086	2.5	1	0.002	0.12	HEP	EUA
6	6	5	0.043	1.2	1	3×10^{-4}	0.13	HEP	EUT
6	8	5	0.057	1.7	0.999	0.003	0.11	HEP	PHA
7	12	7	0.100	2.9	1	0.001	0.14	LYB	EUA
7	6	6	0.05	1.4	1	1×10^{-5}	0.16	LYB	EUT
7	8	7	0.067	1.9	1	1×10^{-5}	0.18	LYB	PHA
12	6	6	0.086	2.5	1	0.002	0.12	EUA	EUT
12	8	8	0.114	3.3	1	1×10^{-4}	0.16	EUA	PHA
6	8	6	0.057	1.7	1	6×10^{-5}	0.15	EUT	PHA

Supplementary File 5b. Summary of significant co-occurrence pairs in the Andaman Islands (AND). Obs.cooc – observed number of co-occurrences; Prob.cooc – probability of co-occurrence; Exp.cooc – expected number of co-occurrences; p_{lt}– probability that the two species would co-occur at a frequency less than the observed number of co-occurrence sites if the two species were distributed randomly (independently) of one another; p_{gt} – probability of co-occurrence at a frequency greater than the observed frequency; Std.eff – standardized effect size. In this case, all are positive co-occurrences.

No. sites with Sp1	No. sites with Sp2	Obs. cooc.	Prob. cooc.	Exp. cooc.	p_{lt}	p_{gt}	Std.eff	Sp1 name	Sp2 name
11	11	11	0.617	8.6	1	0.002	0.17	COS	GEV
5	8	5	0.204	2.9	1	0.028	0.15	SPM	PHA
5	7	5	0.179	2.5	1	0.011	0.18	PSA	LYB
5	6	5	0.153	2.1	1	0.003	0.21	PSA	EUT
5	8	5	0.204	2.9	1	0.028	0.15	PSA	PHA
6	7	6	0.214	3	1	0.002	0.21	CNA	COB
7	7	6	0.250	3.5	0.999	0.015	0.18	COB	LYB
4	7	4	0.143	2	1	0.035	0.14	GEM	LYB
6	6	5	0.184	2.6	0.999	0.016	0.17	HEP	EUT
7	6	6	0.214	3	1	0.002	0.21	LYB	EUT
7	8	7	0.286	4	1	0.002	0.21	LYB	PHA
6	8	6	0.245	3.4	1	0.009	0.19	EUT	PHA

Supplementary File 5c. Summary of significant co-occurrence pairs in the Nicobar Islands (NIC). Obs.cooc – observed number of co-occurrences; Prob.cooc – probability of co-occurrence; Exp.cooc – expected number of co-occurrences; p_{lt}– probability that the two species would co-occur at a frequency less than the observed number of co-occurrence sites if the two species were distributed randomly (independently) of one another; p_{gt} – probability of co-occurrence at a frequency greater than the observed frequency; Std.eff – standardized effect size. Shaded species pairs show significant negative co-occurrences.

No. sites with Sp1	No. sites with Sp2	Obs. cooc.	Prob. cooc	Exp. cooc	p_{lt}	p_{gt}	Std.eff	Sp1 name	Sp2 name
5	8	0	0.178	2.7	0.007	1	-0.18	BRD	BRR
5	8	5	0.178	2.7	1	0.019	0.15	BRD	CYA
5	6	5	0.133	2	1	0.002	0.20	BRD	GES
5	3	3	0.067	1	1	0.022	0.13	BRD	EUR
5	9	1	0.200	3	0.047	0.998	-0.13	BRD	ERU
5	9	0	0.200	3	0.002	1	-0.20	BRD	PTN
5	9	0	0.200	3	0.002	1	-0.20	BRD	SPM
8	8	2	0.284	4.3	0.032	0.999	-0.15	BRR	CYA
8	6	0	0.213	3.2	0.001	1	-0.21	BRR	GES
8	9	8	0.320	4.8	1	0.001	0.21	BRR	ERU
8	9	8	0.320	4.8	1	0.001	0.21	BRR	PTN
8	9	8	0.320	4.8	1	0.001	0.21	BRR	SPM
6	3	3	0.080	1.2	1	0.044	0.12	GES	EUR
6	9	1	0.240	3.6	0.011	0.999	-0.17	GES	ERU
6	9	0	0.240	3.6	$2{\times}10^{-4}$	1	-0.24	GES	PTN
6	9	0	0.240	3.6	$2{\times}10^{-4}$	1	-0.24	GES	SPM
3	9	0	0.120	1.8	0.044	1	-0.12	EUR	PTN
3	9	0	0.120	1.8	0.044	1	-0.12	EUR	SPM
9	9	8	0.360	5.4	0.999	0.011	0.17	ERU	PTN
9	9	8	0.360	5.4	0.999	0.011	0.17	ERU	SPM
9	9	9	0.360	5.4	1	$2{\times}10^{-4}$	0.24	PTN	SPM

Supplementary File 6. Models of species abundance distribution fitted to the Andaman & Nicobar Islands lizard communities through maximum likelihood based fitting procedure. Models are arranged in descending order based on Akaike Information Criteria.

Model	Fixed parameters	Coefficients	Estimate	Standard error	z	P	Log likelihood	df	AIC	dAIC
**Andaman Islands (AND)**										
Pareto distribution	Scale=22	Shape	0.57	0.22	2.65	8.15e-3 **	-44.72	1	91.40	0.00
Broken stick	N=1788 S=7	na	na	na	na	na	-45.79	0	91.60	0.10
Lognormal distribution	None	meanlog	4.83	0.49	9.77	< 2.20e-16 ***	-45.65	2	95.30	3.90
		sdlog	1.31	0.35	3.74	1.83e-4 ***				
Poisson-lognormal distribution	None	μ	4.83	0.50	9.70	< 2.2e-16***	-45.68	2	95.40	3.90
		σ	1.31	0.36	3.66	2.51e-4***				
Weibull distribution	None	Shape	0.88	0.27	3.29	9.93e-3 ***	-45.71	2	95.40	4.00
		Scale	240.20	108.78	2.21	2.72e-2 *				
Volkov neutral biodiversity distribution	J=1788	Θ	1.82	3.68	0.50	0.62	-46.61	2	97.20	5.80
		m	0.01	0.01	0.43	0.67				
Metacommunity zero-sum multinomial distribution	J=1788	Θ	1.16	0.90	1.29	0.20	-48.43	1	98.90	7.40
Log series	N=1788	α	0.93	1.03	0.90	0.37	-48.92	1	99.80	8.4
Power-discreet distribution	None	s	1.19	0.07	16.82	< 2.20e-16***	-52.63	1	107.30	15.80
Geometric series	S=7	k	0.43	0.01	46.63	< 2.20e-16***	-2647.28	1	5296.60	5205.10
Zipf-Mandelbrot distribution (RAD)	N=7	s	58.51	27.19	2.15	3.14e-2*	-2649.54	2	5571.70	5480.30
		v	99.25	47.24	2.10	3.57e-2*				
Zipf distribution (RAD)	N=7	s	1.28	0.04	36.21	< 2.20e-16 ***	-2784.85	1	5571.70	5480.30
**Little Andaman Island (LAND)**										
Pareto distribution	Scale=20	Shape	0.48	0.18	2.65	8.15e-3 **	-47.80	1	97.60	0.00
Broken stick	N=3630 S=7	None	na	na	na	na	-50.98	0	102.00	4.40
Lognormal distribution	None	meanlog	5.09	0.64	7.91	2.55e-15 ***	-49.28	2	102.60	5.00
		sdlog	1.70	0.45	3.74	1.83e-4 ***				
Poisson-lognormal distribution	None	μ	5.08	0.66	7.72	1.19e-14 ***	-49.30	2	102.60	5.00
		σ	1.72	0.48	3.59	3.33e-4 ***				
Volkov neutral biodiversity	J=3630	Θ	0.22	1.82	0.12	0.91	-49.44	2	102.90	5.30

distribution		m	0.03	0.05	0.62	0.54				
Weibull distribution	None	Shape	0.65	0.20	3.33	8.78e-4 ***	-49.56	2	103.10	5.50
		Scale	383.98	236.19	1.63	0.10				
Metacommunity zero-sum multinomial distribution	J=3630	Θ	1.05	na	na	na	-50.83	1	103.70	6.10
Log series	N=3630	α	0.84	0.97	0.86	0.39	-51.34	1	104.70	7.10
Power-discreet distribution	None	s	1.18	0.07	17.51	< 2.20e-16 ***	-54.75	1	111.50	13.90
Geometric series	S=7	k	0.517	0.007	79.119	< 2.20e-16 ***	-4723.02	1	9448.00	9350.40
Zipf-Mandelbrot distribution (RAD)	N=7	s	131.07	na	na	na	-4726.19	2	9456.40	9358.80
		v	177.70	na	na	na				
Zipf distribution (RAD)	N=7	s	1.555	0.026	58.920	< 2.20e-16 ***	-5068.42	1	10138.80	10041.20
**South Andaman Island (SAND)**										
Pareto distribution	Scale=13	Shape	0.50	0.20	2.45	0.01 *	-37.57	1	77.10	0.00
Broken stick	N=1415 S=6	None	na	na	na	na	-39.42	0	78.80	1.70
Lognormal distribution	None	meanlog	4.57	0.58	7.90	2.71e-15 ***	-38.01	2	80.00	2.90
		sdlog	1.42	0.41	3.46	5.32e-4 ***				
Poisson-lognormal distribution	None	μ	4.57	0.59	7.77	7.78e-15 ***	-38.03	2	80.10	2.90
		σ	1.42	0.43	3.33	8.69e-4 ***				
Weibull distribution	None	Shape	0.76	0.24	3.20	1.37e-3 **	-38.32	2	80.60	3.50
		Scale	196.73	112.58	1.75	0.08				
Gamma distribution	None	Shape	0.68	0.33	2.04	0.04 *	-38.43	2	80.90	3.70
		Rate	0.00	0.00	1.43	0.15				
Volkov neutral biodiversity distribution	J=1415	Θ	0.32	2.28	0.14	0.89	-38.51	2	81.00	3.90
		m	0.02	0.07	0.35	0.72				
Metacommunity zero-sum multinomial distribution	J=1415	Θ	1.00	na	na	na	-39.76	1	81.50	4.40
Log series	N=1415	α	0.80	0.96	0.83	0.40	-40.30	1	82.60	5.40
Power-discreet distribution	None	s	1.20	0.08	14.92	< 2.20e-16 ***	-43.21	1	88.40	11.30
Zipf-Mandelbrot distribution (RAD)	N=6	s	4.37	1.31	3.32	8.92e-4 ***	-1608.00	2	3220.00	3142.80
		v	2.65	1.45	1.83	6.76e-02				

Geometric series	S=6	k	0.58	0.01	53.07	< 2.20e-16 ***	-1614.88	1	3231.8<br>0	3154.60
Zipf distribution (RAD)	N=6	s	1.86	0.05	37.98	< 2.20e-16 ***	-1615.74	1	3233.5<br>0	3156.30
**Rutland Island (RUT)**										
Broken Stick	N = 1320<br>S = 5	na	na	na	na	na	-33.11	0	66.20	0.00
Pareto distribution	Scale = 40	Shape	0.71	0.32	2.24	0.03	-32.14	1	66.30	0.10
Lognormal	None	meanlog<br>sdlog	5.09<br>0.97	0.43<br>0.31	11.76<br>3.16	< 2.20e-16<br>0.00	-32.39	2	68.80	2.50
Poisson lognormal	None	µ<br>σ	5.09<br>0.96	0.43<br>0.31	11.74<br>3.12	< 2.20e-16<br>0.00	-32.40	2	68.80	2.60
Gamma distribution	None	shape<br>rate	1.17<br>0.00	0.66<br>0.00	1.77<br>1.43	0.08<br>0.15	-32.84	2	69.70	3.50
Weibull distribution	None	shape<br>scale	1.03<br>267.65	0.34<br>123.51	3.02<br>2.17	0.00<br>0.03	32.88	2	69.80	3.50
Volkov distribution	J = 1320	Θ<br>m	0.22<br>0.01	2.16<br>0.02	0.10<br>0.65	0.92<br>0.51	-33.94	2	71.90	5.70
Metacommunity zero-sum multinomial	J = 1320	Θ	1.00	na	na	na	-35.70	1	73.40	7.20
Logseries	N = 1320	α	0.66	0.87	0.76	0.45	-36.26	1	74.50	8.30
Power discreet distribution	None	s	1.18	0.08	14.80	< 2.20e-16	-39.11	1	80.20	14.00
Zipf distribution	N= 5	s	1.63	0.05	31.62	< 2.20e-16	-1540.43	1	3082.9<br>0	3016.60
Geometric series	S = 5	k	0.53	0.01	42.28	< 2.20e-16	-1577.53	1	3157.1<br>0	3090.80
**North Andaman (NAND)**										
Pareto distribution	Scale = 50	Shape	1.30	0.53	2.45	0.01	-32.54	1	67.10	0.00
Broken Stick	N =925<br>S = 6	na	na	na	na	na	-36.08	0	72.20	5.10
Lognormal	None	meanlog<br>sdlog	4.68<br>0.82	0.34<br>0.24	13.97<br>3.46	<2.20e-16<br>0.00	-35.43	2	74.90	7.80
Poisson lognormal	None	µ<br>σ	4.69<br>0.82	0.34<br>0.24	13.85<br>3.42	<2.20e-16<br>0.00	-35.47	2	74.90	7.90

Gamma distribution	None	shape<br>rate	1.56<br>0.01	0.82<br>0.01	1.90<br>1.61	0.06<br>0.11	-35.92	2	75.80	8.70
Weibull distribution	None	shape<br>scale	1.24<br>166.34	0.39<br>58.31	3.18<br>2.85	0.00<br>0.00	-36.02	2	76.00	9.00
Volkov distribution	J = 925	Θ<br>m	1.15<br>0.01	2.75<br>0.02	0.42<br>0.56	0.68<br>0.58	-38.06	2	80.10	13.00
Metacommunity zero-sum multinomial	J = 925	Θ	1.10	na	na	Na	-40.11	1	82.20	15.10
Logseries	N = 925	α	0.86	1.00	0.86	0.39	-40.62	1	83.20	16.20
Power discreet distribution	None	s	1.19	0.08	15.21	<2.20e-16	-44.04	1	90.10	23.00
Geometric series	S = 6	k	0.39	0.01	27.02	<2.20e-16	-1390.50	1	2783.00	2715.90
Zipf-Mandelbrodt distribution	N = 6	s<br>v	7.57<br>12.49	6.19<br>12.47	1.22<br>1.00	0.22<br>0.32	-1389.60	2	2783.20	2716.10
Zipf distribution	N=6	s	1.14	0.05	22.06	<2.20e-16	-1406.98	1	2816.00	2748.90
**Neil Island (NEIL)**										
Broken Stick	N = 2700<br>S = 5	na	na	na	na	na	-36.48292	0	73.00	0.00
Pareto distribution	Scale = 50	Shape	0.57	0.26	2.24	0.03	-36.09	1	74.20	1.20
Gamma distribution	None	shape<br>rate	0.92<br>0.00	0.51<br>0.00	1.81<br>1.38	0.07<br>0.17	-36.45	2	76.90	3.90
Weibull distribution	None	shape<br>scale	0.94<br>526.11	0.34<br>263.17	2.81<br>2.00	0.01<br>0.05	-36.44	2	76.90	3.90
Lognormal	None	meanlog<br>sdlog	5.66<br>1.24	0.55<br>0.39	10.21<br>3.16	< 2.20e-16<br>0.00	-36.47	2	76.90	4.00
Poisson lognormal	None	μ<br>σ	5.66<br>1.24	0.55<br>0.39	10.20<br>3.14	< 2.20e-16<br>0.00	-36.47	2	76.90	4.00
Volkov distribution	J = 2700	Θ<br>m	1.12<br>0.00	4.18<br>0.01	0.27<br>0.36	0.79<br>0.72	-37.32	2	78.60	5.70
Metacommunity zero-sum multinomial	J = 2700	Θ	1.00	na	na	na	-38.99	1	80.00	7.00
Logseries	N = 2700	α	0.59	0.82	0.72	0.47	-39.55	1	81.10	8.10
Power discreet distribution	None	s	1.16	0.07	16.08	< 2.20e-16	-42.44	1	86.90	13.90

Geometric series	S = 5	k	0.55	0.01	63.92	< 2.20e-16	-3120.96	1	6243.90	6170.90
Zipf-Mandelbrodt distribution	N = 5	s<br>v	78.10<br>95.20	62.36<br>77.89	1.25<br>1.22	0.21<br>0.22	-3121.70	2	6247.40	6174.40
Zipf distribution	N = 5	s	1.60	0.04	44.73	< 2.20e-16	-3186.94	1	6375.90	6302.90
**Camorta Island (CAM)**										
Pareto distribution	Scale = 25	Shape	0.74	0.37	2.00	0.05	-23.46	1	48.90	0.00
Broken Stick	N = 750<br>S = 4	na	na	na	na	na	-25.30	0	50.60	1.70
Lognormal	None	meanlog<br>sdlog	4.57<br>1.16	0.58<br>0.41	7.90<br>2.83	0.00<br>0.00	-24.53	2	53.10	4.10
Poisson lognormal	None	μ<br>σ	4.57<br>1.16	0.58<br>0.42	7.83<br>2.78	0.00<br>0.01	-24.55	2	53.10	4.20
Weibull distribution	None	shape<br>scale	0.88<br>175.18	0.34<br>105.27	2.63<br>1.66	0.01<br>0.10	-24.88	2	53.80	4.80
Gamma distribution	None	shape<br>rate	0.88<br>0.00	0.54<br>0.00	1.62<br>1.23	0.10<br>0.22	-24.91	2	53.80	4.90
Volkov distribution	J = 750	Θ<br>m	0.20<br>0.02	2.62<br>0.04	0.08<br>0.44	0.94<br>0.66	-25.07	2	54.10	5.20
Metacommunity zero-sum multinomial	J = 750	Θ	1.00	na	na	na	-26.16	1	54.30	5.40
Logseries	N = 750 S = 4	α	0.55	0.80	0.69	0.49	-26.73	1	55.50	6.50
Power discreet distribution	None	s	1.20	0.10	12.18	< 2.20e-16	-28.81	1	59.60	10.70
Zipf distribution	N = 4	s	2.18	0.09	24.78	< 2.20e-16	-615.10	1	1232.20	1183.30
Zipf-Mandelbrodt distribution	N = 4	s<br>v	29.40<br>22.48	na<br>na	na<br>na	na<br>na	-621.09	2	1246.20	1197.30
Geometric series	S = 4	k	0.70	0.02	44.00	< 2.20e-16	-622.59	1	1247.20	1198.20
**Great Nicobar Island (GNI)**										
Pareto distribution	Scale=30	Shape	1.22	0.54	2.24	0.03 *	-25.13	1	52.30	0.00
Broken stick	N=460 S=5	None	na	na	na	na	-27.37	0	54.70	2.50

Lognormal distribution	None	meanlog<br>sdlog	4.22<br>0.78	0.35<br>0.25	12.10<br>3.16	< 2.20e-16 ***<br>0.002 **	-26.97	2	57.90	5.70
Poisson-lognormal distribution	None	μ<br>σ	4.23<br>0.78	0.35<br>0.25	12.00<br>3.07	< 2.20e-16 ***<br>0.002 **	-27.01	2	58.00	5.80
Gamma distribution	None	Shape<br>Rate	1.82<br>0.02	1.06<br>0.01	1.71<br>1.49	0.09<br>0.14	-27.16	2	58.30	6.10
Weibull distribution	None	Shape<br>Scale	1.38<br>101.41	0.48<br>34.79	2.87<br>2.91	0.004 **<br>0.004 **	-27.24	2	58.50	6.20
Volkov neutral biodiversity distribution	J=460	Θ<br>m	0.89<br>0.02	2.88<br>0.03	0.31<br>0.59	0.76<br>0.55	-29.05	2	62.10	9.90
Metacommunity zero-sum multinomial distribution	J=460	Θ	1.00	na	na	na	-30.63	1	63.30	11.00
Log series	N=460	α	0.78	0.97	0.81	0.42	-31.16	1	64.30	12.10
Power-discreet distribution	None	s	1.21	0.09	12.84	< 2.20e-16 ***	-33.93	1	69.90	17.60
Geometric series	S=5	k	0.43	0.02	19.14	< 2.20e-16 ***	-618.25	1	1238.50	1186.20
Zipf-Mandelbrot distribution (RAD)	N=5	s<br>v	7.38<br>10.49	11.14<br>19.51	0.66<br>0.54	0.51<br>0.59	-617.93	2	1239.90	1187.60
Zipf distribution (RAD)	N=5	s	1.20	0.08	14.98	< 2.20e-16 ***	-622.42	1	1246.80	1194.60

Supplementary File 7. Histogram of simulated variance ratios for (a) South Andaman, (b) Great Nicobar, (c) Tarmugli, & (c) Car Nicobar. The red vertical line indicates the observed variance ratio, the long-dash lines indicate 95% 1-tailed cut points, and the short-dash lines indicate 95% 2-tailed cut points or 95% confidence interval for the null model.

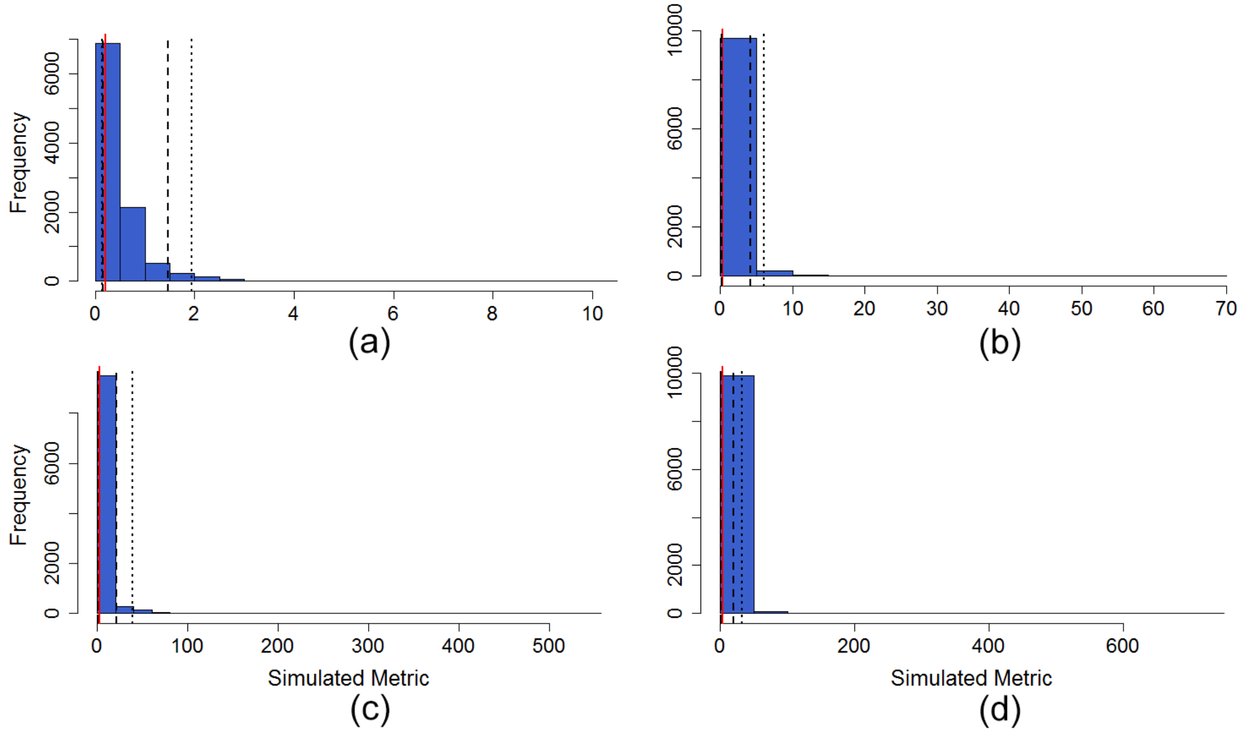

Supplement: Supplementary file 1 — Supplementary information [file 41598_2018_30427_MOESM1_ESM.pdf]
